# Up-regulation of Hsp72 and keratin16 mediates wound healing in streptozotocin diabetic rats

**DOI:** 10.1186/s40659-015-0044-5

**Published:** 2015-10-01

**Authors:** Rasha R. Ahmed, Ayman Mahmoud, Osama M. Ahmed, Ali Metwalli, Hossam Ebaid

**Affiliations:** Cell Biology and Histology Division, Zoology Department, Faculty of Science, Beni-Suef University, Beni-Suef, Egypt; Physiology Division, Zoology Department, Faculty of Science, Beni-Suef University, Beni-Suef, Egypt; Department of Food Science, College of Agriculture and Food Science, King Saud University, Riyadh, Saudi Arabia; Department of Dairy, Faculty of Agriculture, El-Minia University, El-Minia, Egypt; Department of Zoology, College of Science, King Saud University, P.O. Box 2455, Riyadh, 11451 KSA; Department of Zoology, Faculty of Science, El-Minia University, El-Minia, Egypt

**Keywords:** Diabetic model, Whey protein, Heat shock protein-72, Keratin16, Wound healing

## Abstract

**Background:**

Impaired wound healing is a complication of diabetes and a serious problem in clinical practice. We previously found that whey protein (WP) was able to regulate wound healing normally in streptozotocin (STZ)-diabetic models. This subsequent study was designed to assess the effect of WP on heat shock protein-72 (Hsp72) and keratin16 (Krt16) expression during wound healing in diabetic rats.

**Methods:**

WP at a dosage of 100 mg/kg of body weight was orally administered daily to wounded normal and STZ-diabetic rats for 8 days.

**Results:**

At day 4, the WP-treated diabetic wound was significantly reduced compared to that in the corresponding control. Diabetic wounded rats developed severe inflammatory infiltration and moderate capillary dilatation and regeneration. Treated rats had mild necrotic formation, moderate infiltration, moderate to severe capillary dilatation and regeneration, in addition to moderate epidermal formation. Hsp72 and Krt16 densities showed low and dense activity in diabetic wounded and diabetic wounded treated groups, respectively. At day 8, WP-treatment of diabetic wounded animals revealed great amelioration with complete recovery and closure of the wound. Reactivity of Hsp72 and Krt16 was reversed, showing dense and low, or medium and low, activity in the diabetic wounded and diabetic wounded treated groups, respectively. Hsp72 expression in the pancreas was found to show dense reactivity with WP-treated diabetic wound rats.

**Conclusion:**

This data provides evidence for the potential impact of WP in the up-regulation of Hsp72 and Krt16 in T1D, resulting in an improved wound healing process in diabetic models.

## Background

Impaired diabetic wound healing occurs as a consequence of excessive reactive oxygen species (ROS) and inflammatory cytokine production [1–3]. Intracellular heat shock proteins (Hsps) are upregulated in cells subjected to stressful stimuli, including inflammation and oxidative stress, and exert a protective effect against hypoxia, excess oxygen radicals, endotoxin, infections and fever [4]. Recent studies have confirmed that Hsps support antioxidant [5], anti-inflammatory [6] or immune enhancing [7] therapies in sepsis. Park et al. [8] found that Hsp72 functions as an endogenous inhibitor of apoptosis signal-regulating kinase 1. It plays a role in many cellular activities, including prevention of cell death initiated by various apoptotic stresses, such as ionizing irradiation and TNF-a [9, 10], by suppressing apoptotic signalling pathways, including caspase cascades [11] and the JNK signalling pathway [12].

Keratins are the most prominent cytoskeletal proteins in keratinocytes [13]. Keratin 16 (Krt16) is usually referred to as an activation- and hyperproliferation-associated keratin [14]. Although, Krt16 impacts cell migration by interacting with Src kinase [15], its induction significance in response to environmental stressors in epithelial cancers and in chronic inflammatory disorders [16] is largely unknown. Juliane et al. [17] showed that when the epidermal barrier is experimentally challenged by acute pro-inflammatory and mechanical stimuli, keratinocytes lacking Krt16 fail to regulate the production of innate danger signals properly, and over-activate the expression of cytokines and other regulators of skin barrier function.

Whey protein (WP) has been shown to be able to regulate impaired wound healing normally [18]. Here we aimed to find a correlation between the achievement of normal healing in diabetic wounds by WP and the regulation of two important markers, Hsp72 and Krt16.

## Methods

After shaving and disinfecting the rat dorsal skin surface, a skin wound site was made using a Biopsy Punch (5 mm in the diameter).

Rats, at the end of each tested period, were euthanatized and 10 × 10 mm of skin including the wound site and pancreas were excised then fixed in 10 % neutral formalin solution. After washing with distilled water, the paraffin embedding specimens were developed in the routine method. Immunohistochemical, and hematoxylin & eosin (HE) staining were carried out on 4 μm-thick sections.

### Measurement of wound diameter and closure

The wound diameter was measured on days 1, 4 and 8 after incision and percentage of wound closure was calculated using the following formula: wound closure rate on day X (%) = [(wound diameter on day 0 − wound diameter on day X)/(wound diameter on day 0)] × 100.

### Ethical approval and preparation of un-denatured camel milk whey proteins

Camel milk was obtained from a camel breed (Majaheem) from the Najd region (Alazeria farm; GPS: 300 02 47/300 02 27) in Saudi Arabia. Specific permissions were not required for activities in this private farm. This study did not involve endangered or protected species. Regarding experimental animals, all procedures were conducted in accordance with the standards set forth in the guidelines for the care and use of experimental animals by the Committee for the Purpose of Control and Supervision of Experiments on Animals and the National Institutes of Health. The study protocol (care and handling of experimental animals) was approved by the Animal Ethics Committee of the Zoology Department in the College of Science at King Saud University.

The milk was skimmed by centrifugation at 5000*g* for 20 min using an IEC Model K centrifuge (Boston, USA). Skim milk was acidified to pH 4.3 using 1 M of HCl. The precipitated casein was removed by centrifugation, and the supernatant containing the whey protein was saturated with ammonium sulfate (70 % saturation) and incubated overnight at 4 °C. The precipitated whey proteins were collected by centrifugation and dialyzed against distilled water for 48 h at 4 °C using a Spectra/Pro^®^ Membrane, MWCO 6000-8000 Da. The obtained dialyzate was lyophilized using a Unitop 600 SL, (Virtis Company, Gardiner, New York 12525 USA) and were kept at −20 °C until use. The dialyzate containing un-denatured whey proteins were freeze-dried and refrigerated until use.

### Diabetic models

Diabetes was induced by a single injection of freshly dissolved STZ (60 mg/kg of body weight; Sigma, USA) in a 0.1 mol/l citrate buffer (pH 4.5) into the peritoneum. Control rats were injected with citrate buffer. Seven days after STZ injection, the rats were screened for serum glucose levels. Rats with a serum glucose level ≥200 mg/dl after 2 h of glucose intake were considered diabetic and selected for further studies.

### Experimental design

The supplemented volume for all groups was constant and did not exceed 250 μl per dosage per day. The optimal dose of WP was determined in our laboratory on the basis of the LD50 and several established studies and parameters. The animals were allocated into 6 groups of 12 animals each, assigned as follows:

1. Uninjured control group that were orally supplemented with distilled water (250 μl/rat/day).
2. Wounded non-diabetic group with daily administration of the vehicle (250 μl/rat/day), 1 % carboxymethyl cellulose (CMC), by gastric intubation for 4 days or by gastric intubation for 8 days.
3. Wounded non-diabetic group with daily administration of WP at 100 mg/kg of body weight (250 μl/rat/day), dissolved in 1 % CMC, by gastric intubation either for 4 days or for 8 days.
4. Uninjured diabetic group (non-wounded diabetic: non-wounded D) that were orally supplemented with distilled water (250 μl/rat/day).
5. Wounded diabetic group with daily administration of 1 % CMC (250 μl/rat/day) by gastric intubation for 4 days or by gastric intubation for 8 days.
6. Wounded diabetic group with daily treatment of WP at 100 mg/kg of body weight (250 μl/rat/day) by gastric intubation either for 4 days or for 8 days.

### Histological analyses

After fixation with 4 % paraformaldehyde for 24 h at room temperature, the specimens were embedded in paraffin and sectioned in a plane perpendicular to the incision. Sections 5 μm thick were mounted on slides, dewaxed, rehydrated to distilled water, and stained with HE. For each group, three sections of three different animals were randomly selected for histological evaluation. The mean value was used for statistical comparison.

### Immunohistochemical study

The streptavidin–biotin-peroxidase technique was used for tests with anti-Hsp72 (Catalog No. SPA-810, Stressgen, USA), five-micrometer-thick sections were de-waxed and rehydrated in a descending series of alcohols. Antigen retrieval by microwave and citrate buffer (pH 6) was performed, using the procedure specified by the antibody manufacturer. Slides were subjected to endogenous tissue peroxidase blocking. Incubation was performed with the primary antibody at a dilution of 1:1000 in PBS/0.1 % Tween. The samples were then incubated with a biotinylated swine-anti-rabbit/goat antibody, as well as a streptavidin–biotin-peroxidase conjugate (LSAB System, Dako^®^, Carpenteria, CA, USA) for 30 min each. The reaction was then revealed by diaminobenzidine (Dako^®^), and the sections were dehydrated in an increasing series of alcohols, immersed in xylol, and mounted in resin for conventional light microscopy. For the negative control, sections were incubated in a buffer without primary antibody.

Additional immuno-histochemical reactions against cytokeratin 16 were performed. Primary antibodies of cytokeratin 16 (1:500; NeoMarkers^®^, Fremond, CA, USA) were used after antigen retrieval (citric acid in water bath at 100 °C for 45 min for cytokeratin 16). Incubation and reaction of secondary antibodies were the same as those described for Hsp.

### Evaluation of the intensity of immunohistochemical staining

All expression patterns were analyzed by two independent investigators experienced in skin and pancreas histology. The expression densities of Hsp72 in skin and pancreas and krt16 ranged from 0 = no positive cells (undetected), 1 = low density (<10 % positive cells), 2 = medium density (10–25 % positive cells), 3 = dense (26–70 % positive cells), 4 = very dense (71 % positive cells) as described previously by Souil et al. [19]^.^

### Statistical analysis

The data of wound diameter were analyzed using one-way analysis of variance (ANOVA) followed by LSD test to compare various groups with each others according our previously publication [2].

## Results

### Wound closure

**Fig. 1:**
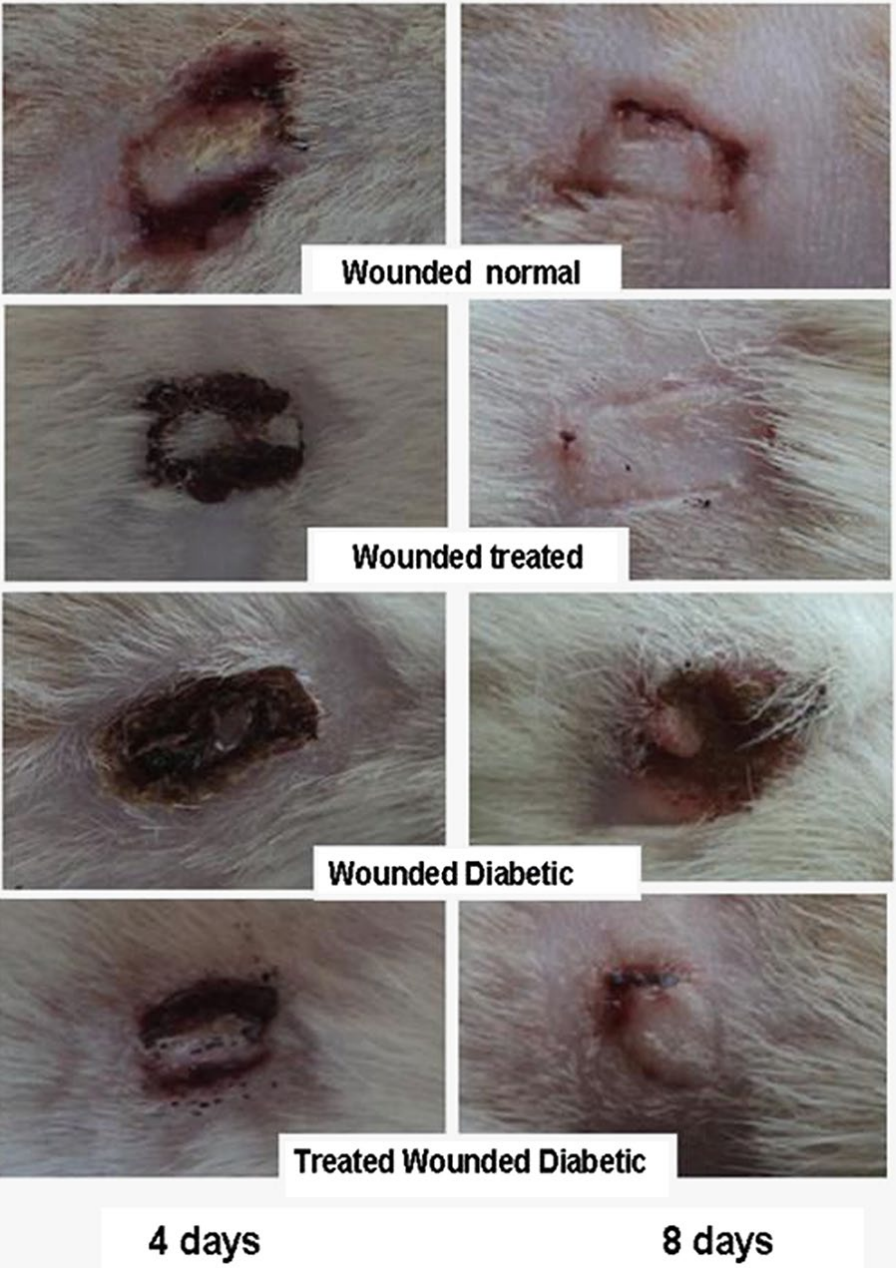
Progress of cutaneous wound healing in control wounded, control wounded treated, diabetic wounded control and diabetic wounded treated groups 4 days (*right column*) and 8 days (*left column*) after incision (from Ebaid et al. [2]). ×4

The diameter of the wound site in each of the groups was measured 1, 4 and 8 days after the incision (Fig. 1; Table 1). In control animals, the diameter of the wound reduced to 4.6 ± 0.13 mm 1 day post-incision and to 3.8 ± 0.1 mm 4 days post-incision and 3 ± 0.1 mm recording wound closure percent of 8.0, 24.0 and 40 % respectively. This closure percent was greatly accelerated after treatment as compared to the control wounded animals where the diameter of the wound reduced to 4.2 ± 0.15 mm 1 day post-incision and to 2.5 ± 0.0 mm 4 days post-incision recording wound closure percent of 16.0 and 50.0 respectively. Eight days post-incision, the wound site was covered by epidermis recording wound closure percent of 100 %. In diabetic animals, the wound site reduced to 4.8 ± 0.1 mm 1 day after experiment, to 4.4 ± 0.13 mm at 4 days after, and to 3.6 ± 0.18 mm 8 days after; the recorded wound closure percent were calculated to be 4.0, 12.0 and 28.0 respectively. In diabetic treated rats, the wound was reduced to 4.4 ± 0.12 mm 1 day post-incision, to 4.0 ± 0.0 mm 4 days after, and to 3.2 ± 0.13 mm 8 days after the operation recording wound closure percent 12.0, 20.0 and 36.0 respectively. The diameter of both normal wounded treated group and diabetic wounded group was significantly decreased at 1, 4 and 8 days after wounding as compared to their corresponding controls.

**Table 1:** Effect of treatment with whey protein on wound diameter and closure percent of normal wounded and diabetic wounded rats

Groups	1 day		4 days		8 days	
	Mean	%	Mean	%	Mean	%
Normal wounded	4.6 ± 0.13^ab^	8	3.8 ± 0.1^ef^	24	3.0 ± 0.1 ^g^	40
Normal wounded treated	4.2 ± 0.15 ^cd^	16	2.5 ± 0.0 ^h^	50	0.0 ± 0.0^i^	100
Diabetic wounded	4.8 ± 0.1^a^	4	4.4 ± 0.13^bc^	12	3.6 ± 0.18^f^	28
Diabetic wounded treated	4.4 ± 0.12^bc^	12	4.0 ± 0.0^de^	20	3.2 ± 0.13 ^g^	36

Data are expressed as mean ± SE. Number of samples in each group is 3

Means which share the same superscript symbol(s) are not significantly different

F-probability: p < 0.001; LSD at the 5 % level = 0.201; LSD at the 1 % level = 0.273

### Histological analyses

**Fig. 2:**
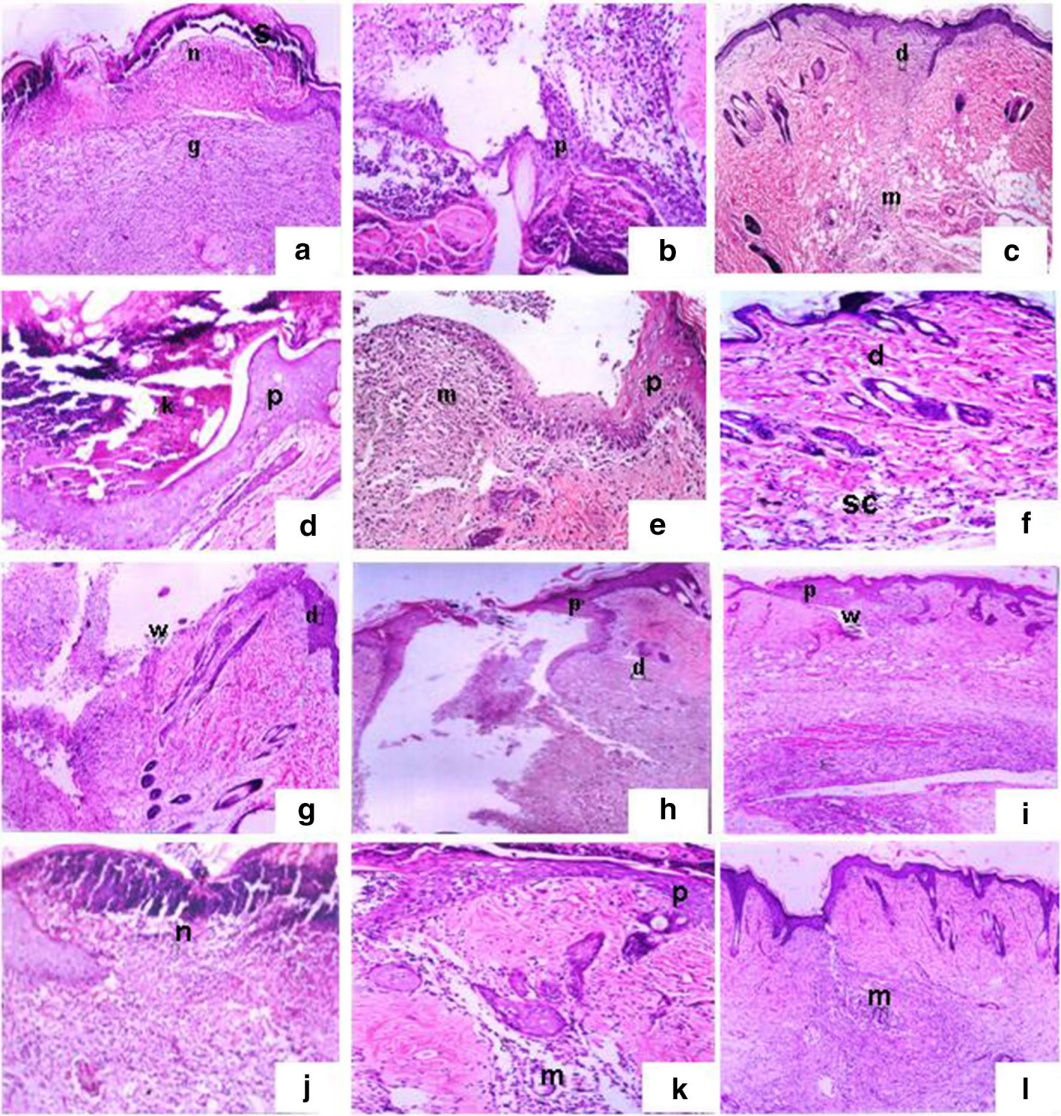
Histological changes during the wound-healing process on the first (*left*), fourth (*centre*) and eighth (*right*) days after incision in control wounded (**a**–**c**), control wounded treated (**d**–**f**), diabetic wounded control (**g**–**i**) and diabetic wounded treated (**j**–**l**) groups; scab (*s*), granulation (*g*), dermis (*d*), wound (*W*), keratinization (*K*), epidermis (*p*), necrosis (*n*) and inflammatory cells (*m*). ×100

**Fig. 3:**
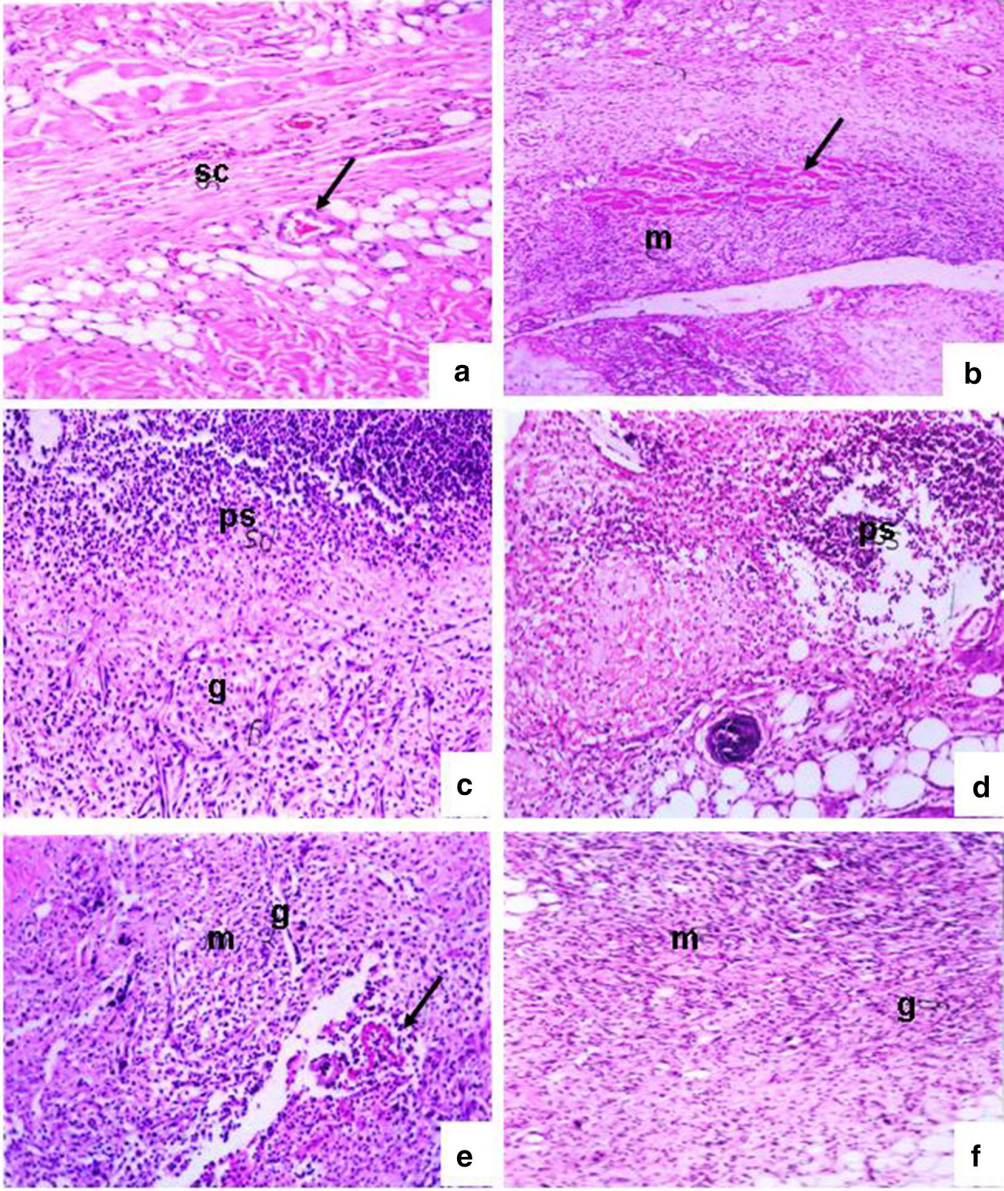
Inflammatory cell response, granulation and blood vessel formation 8 days after wound incision in the control non-wounded group (**a**), control wounded (**b**), diabetic control (**c**), diabetic treated (**d**), diabetic wounded (**e**) and diabetic wounded treated groups (**f**); granulation (*g*), pus cells (*ps*), sub-cutaneous tissue (*sc*), inflammatory cells (*m*) and blood vessels (*arrow*). ×100

The histopathological findings of skin are shown in Figs. 2 and 3.

#### One day after incision

In both wounded non-diabetic animals and diabetic animals that had been wounded but not treated, the wound consisted of a large (scab) uncovered by epidermis. In the wounded treated animals, the wound was filled with necrotic debris and fibrin (crusts) and has proliferating granulation tissue. The dermis showed oedema and vascular congestion. These changes were less apparent in the diabetic un-treated animals than in the non-diabetic control group.

#### Four days after incision

The wound site was diminished due to deep contraction but was still lined by granulation tissue and covered with crusts. The dermis cellularity increased, mainly due to the proliferation of fibroblasts and new matrix deposition. The epidermis was thicker on the margin of the wound (hyperplasia) and was starting to cover the defect. Non-diabetic wounded rats and diabetic animals that had been wounded but not treated developed severe inflammatory infiltration and moderate capillary dilatation and regeneration. All these changes were slightly more evident in diabetic untreated rats compared to the diabetic untreated groups. In contrast, treated rats had mild necrotic formation, moderate infiltration, moderate to severe capillary dilatation and regeneration, in addition to moderate epidermal formation.

#### Eight days after incision:

At day 8, in the wounded animals (both diabetic and non-diabetic) the wound was completely lined by hyperplastic epidermis, but the scar surface was reduced due to wound contraction and matrix deposition. Some immature collagen fibres still remained at the centre. Few inflammatory mononuclear cells remained at the border of the scar. Treatment of both groups revealed a great amelioration of these changes with complete recovery and closure of the wound in the non-diabetic treated group.

#### The histopathological changes in the pancreas

In the diabetic group, a decrease in pancreatic islet numbers and size, atrophy and vacuolation, and invasion of connective tissues in the parenchyma of pancreatic islets were detected after 8 days, but, compared to the diabetic group, these abnormal histological signs dramatically decreased in the group treated with the extract. In contrast, these changes became much more severe in the diabetic wounded groups four and eight days after incision. Treatment of these two latter groups greatly ameliorated these changes.

### Immunohistochemical findings

#### Skin

##### Keratin 16

**Fig. 4:**
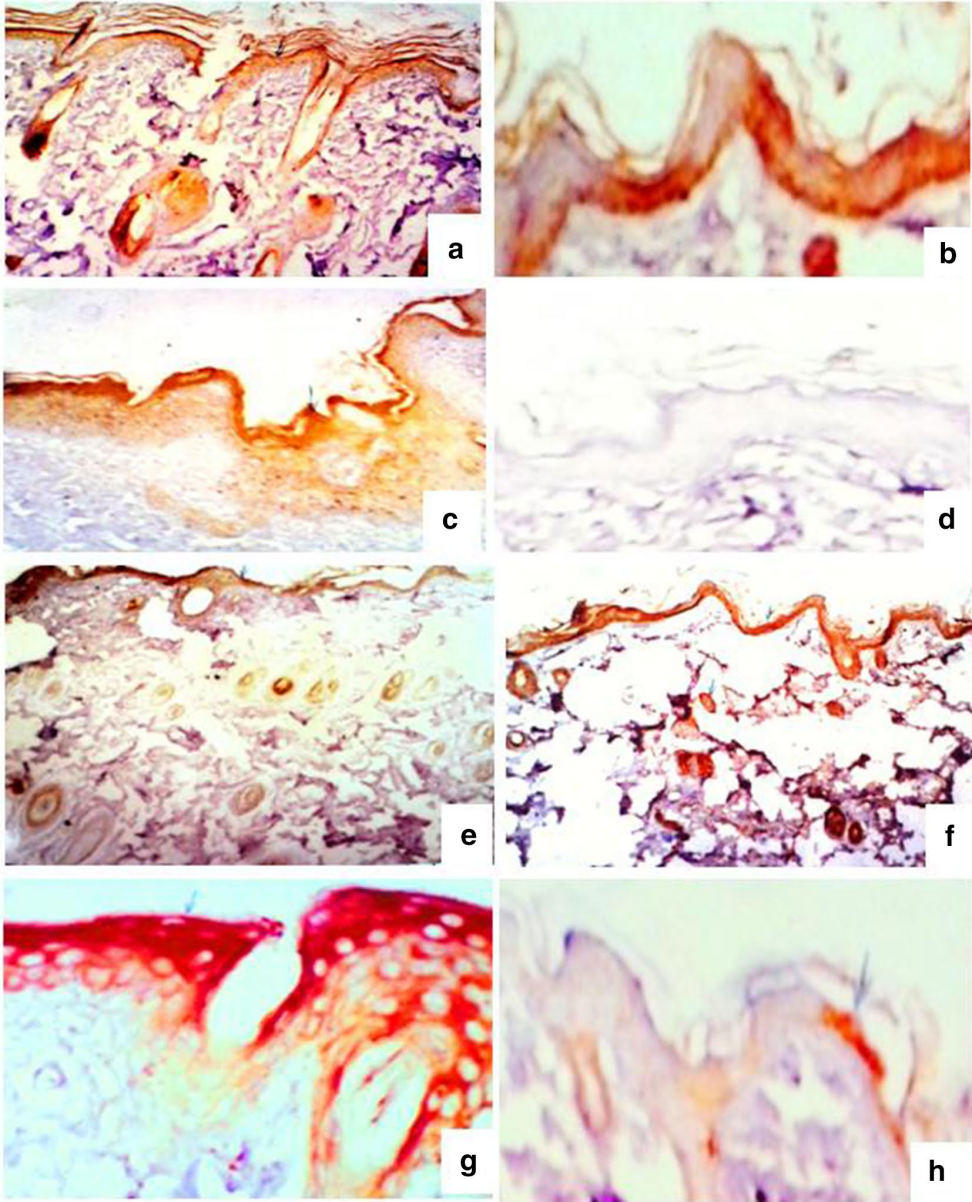
Keratin 16 expression during the wound-healing process four (*left*) and eight (*right*) days after incision in the control wounded (**a**–**b**), control wounded treated (**c**–**d**), diabetic wounded control (**e**–**f**) and diabetic wounded treated (**g**–**h**) groups. (**a**, **c**, **e**, **f** ×100) (**b**, **d**, **g**, **h** ×400)

The intensity of Krt16 in the tested groups and periods are shown in Fig. 4 and Table 2.

**Table 2:** The densities of Krt16 and reactivity in all tested groups and periods

Keratin Krt16	4 days	8 days
Normal wounded	Medium	Low
Normal wounded treated	Medium	None
Diabetic wounded	Low	Medium
Diabetic wounded treated	Dense	Low

Krt16 is not expressed in healthy epidermal skin. Its reaction is restricted to epidermal cells and dermal glands. The reactivity was medium in both normal wounded and normal wounded treated groups while it showed low and dense activity in diabetic wounded and diabetic wounded treated animals 4 days after wound incision.

Eight days after incision, low, no, medium and low reactivity were recorded respectively in normal wounded, normal wounded treated, diabetic wounded and diabetic wounded treated groups.

##### Hsp72

**Fig. 5:**
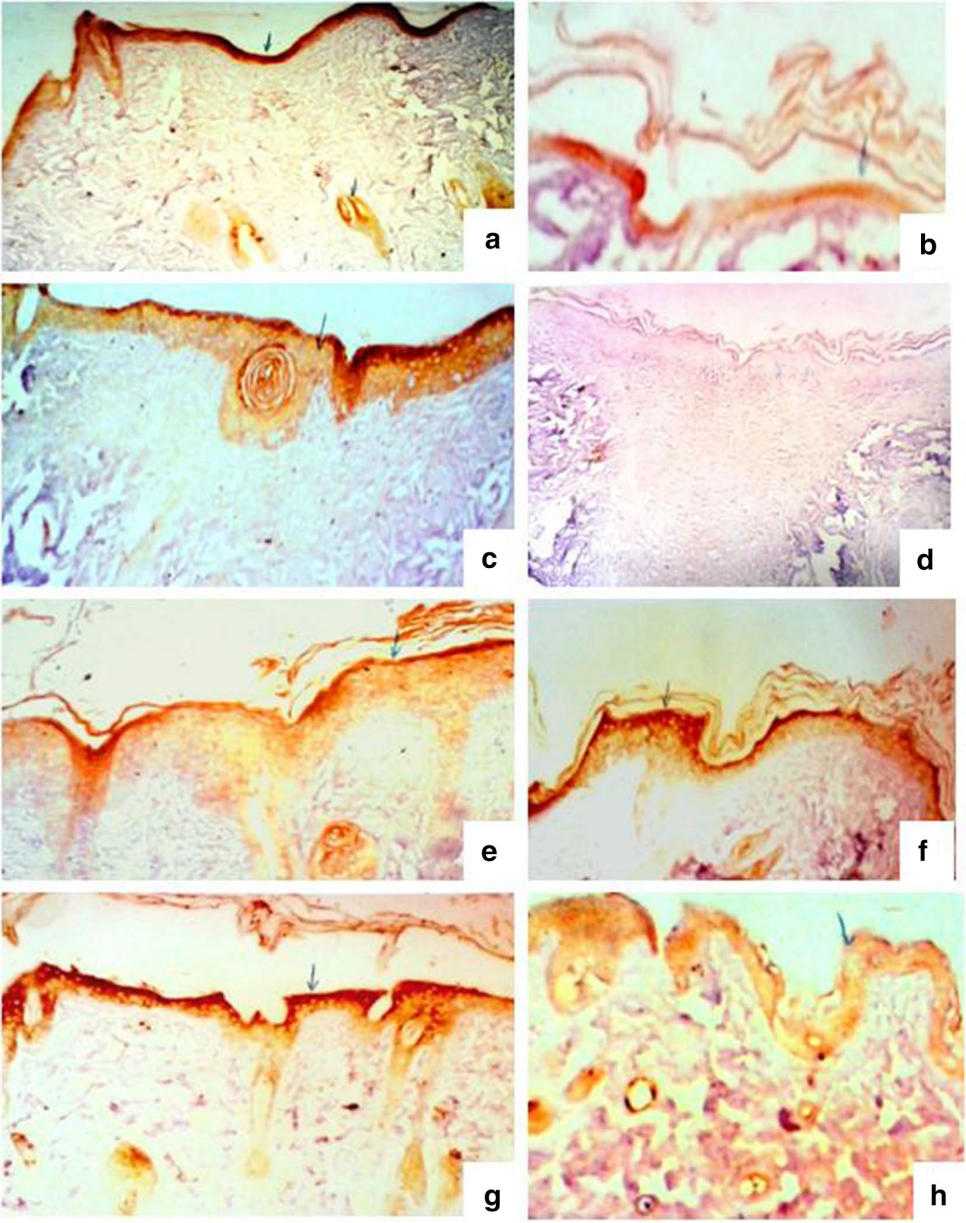
Heat shock protein 72 expression during the wound-healing process four (*left*) and eight (*right*) days after incision in the control wounded (**a**–**b**), control wounded treated (**c**–**d**), diabetic wounded control (**e**–**f**) and diabetic wounded treated (**g**–**h**) groups. ×100

The densities of Hsp72 and reactivity in all tested groups and periods are shown in Fig. 5 and Table 3.

**Table 3:** The densities of Hsp72 and reactivity in all tested groups and periods

HSP72	4 days	8 days
Normal wounded	Dense	Low
Normal wounded treated	Very dense	No
Diabetic wounded	Medium	Dense
Diabetic wounded treated	Dense	Low

**Fig. 6:**
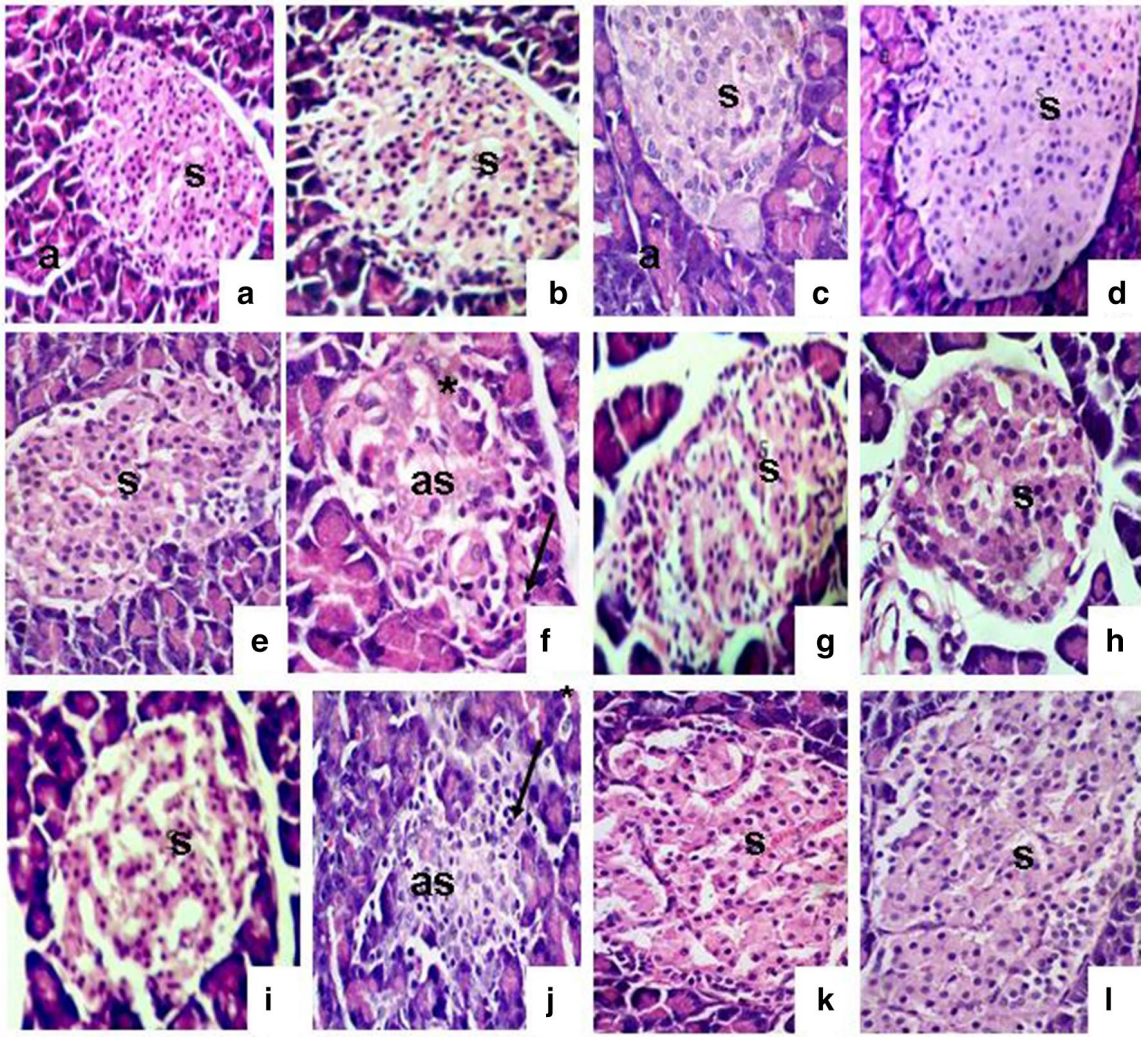
Histological changes during the wound-healing process on the fourth and eighth days, respectively, after incision of the control wounded non-treated (**a**–**b**), control wounded treated (**c**–**d**), diabetic control non-treated (**e**–**f**), diabetic control treated (**g**–**h**), diabetic wounded control (**i**–**j**) and diabetic wounded treated (**k**–**l**) groups; Islands of Langerhans (*s*), atrophied islands (*as*) lymphocytic infiltration (*arrow*) and connective tissue invasion in the parenchyma (*asterisk*). ×400

No difference was recorded in the expression densities of cells positive for Hsp72 in any of the tested groups one day after incision. Four days after wound incision, however, Hsp72 densities were dense, very dense, medium and dense in normal wounded, normal wounded treated, diabetic wounded and diabetic wounded treated groups respectively. This activity was expressed throughout all the layers of the epidermis and dermal glands. Melanocytes, fibroblasts and other epidermal cells were negative, though (Fig. 6).

Eight days after incision, the reactivity in the same kinds of cells were recorded and showed low, no, dense and low activity in normal wounded, normal wounded treated, diabetic wounded and diabetic wounded treated groups, respectively.

#### Pancreas

**Fig. 7:**
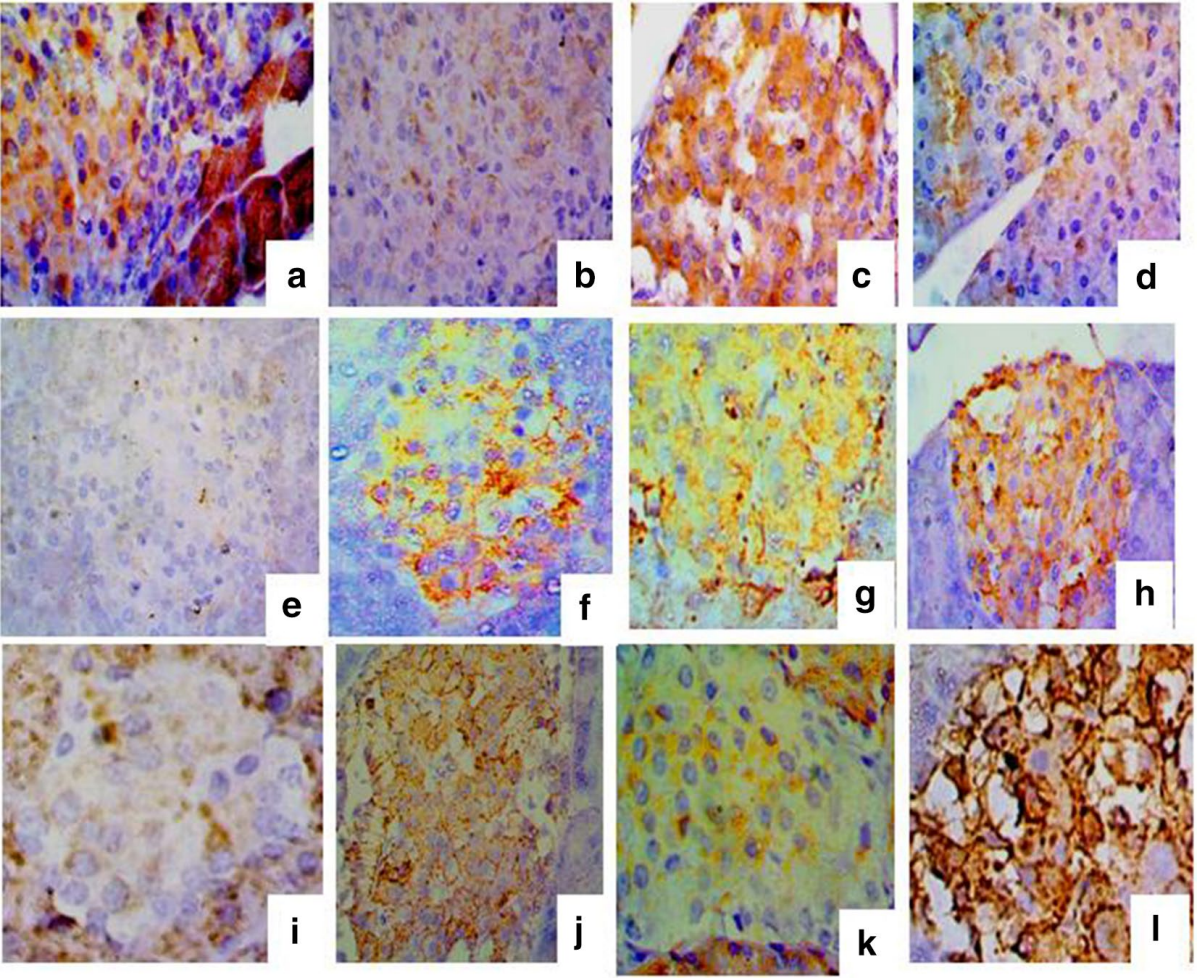
Heat shock protein 72 expression during the wound-healing process four and eight days after incision in the control wounded (**a**–**b**), control wounded treated (**c**–**d**), diabetic non-wounded control (**e**–**f**), diabetic non-wounded treated (**g**–**h**), diabetic wounded control (**i**–**j**) and diabetic wounded treated (**k**–**l**) groups. ×400

The densities of Hsp72 and reactivity in all tested groups and periods are shown in Fig. 7 and Table 4.

**Table 4:** The densities of Hsp72 and reactivity in all tested groups and periods

HSP72	4 days	8 days
Normal wounded	Dense	No
Normal wounded treated	Mild	Low
Diabetic	No	Low
Diabetic treated	Low	Mild
Diabetic wounded	No	Low
Diabetic wounded treated	Low	Dense

Hsp72 expression densities showed no difference in any of the tested groups one day after incision. Four days after wound incision, however, Hsp72 showed dense and mild reactivity in normal wounded and normal wounded treated groups. Hsp72 reactivity was absent or low in the remaining groups, however. The reactivity was mainly recorded in pancreatic islets but it was also noticed in the exocrine part of the control wounded group.

Eight days after incision, the reactivity was noticed in the same types of cells, revealing low reactivity in the normal wounded group and a low response in the diabetic and diabetic wounded animals, as well as low, mild and dense reactivity in the wounded treated, diabetic-treated and diabetic wounded treated groups respectively.

## Discussion

Diabetes decreases immune response capacity, including the suppression of immune cell function and is a major public health concern [20]. Many studies have been performed to assess the potential utility of natural products as immunomodulatory agents to enhance host responses to disease [21]. Although, we have previously proved that WP was able to return wound healing impaired by diabetes to a more normal pattern [1–3], the exact mechanism by which this occurs is still poorly investigated. In this study we have explored the role of Hsp72 and Krt16 in accelerating and normalizing impaired diabetic wound healing in rodent-models.

Hsps play a critical role in the overall process of wound repair [22]. We found that Hsp72, in normal wounds, was highest at day 4, before gradually decreasing during subsequent healing phases. WP was found to induce a similar behaviour of Hsp72 in wounds under the stress of diabetes. Similarly, Hsp72 expression was found to be highest starting from day 3 post-intervention in the spinocellular layer leading to an up-regulation of Hsp47 [23]. Hsp72’s role is as an indicator of cellular stress and injury [22]. Generally, Hsps are chaperone proteins preventing cells from undergoing apoptosis and ensuring their cellular function [24].

Chung et al. [25] used a small molecule activator of Hsp72 to improve insulin sensitivity and inflammation in a genetic mouse model of insulin resistance, and Hsp72 also increases mitochondrial volume and improves metabolic homeostasis in a rat model of T2D [26]. It is likely, therefore, that when WP activates Hsp72, this improves insulin sensitivity and inflammation during diabetic wound healing. Our previous investigation [3] confirmed that WP was able to increase insulin concentration in blood of T1D. Improvements associated with Hsp72 are usually linked with a reduction in JNK1 phosphorylation, as has been previously confirmed by Darren et al. [26]. Hsp72 expression is decreased in T2D [25, 26] because meta-inflammation disrupts insulin signalling. Hsps, therefore, appear to have the potential to inhibit inflammatory kinases. This previous data suggests that improvement of insulin through WP is mediated by Hsp up-regulation. Interestingly, here, Hsp72 in the pancreas was found to show dense reactivity in the WP-treated diabetic wound group.

Furthermore, we found a clear correlation between WP associated up-regulation of Krt16 at day 4 (an indicator of epidermal hyperplasia and keratinocyte migration), and the complete and normal healing of diabetic wounds. In contrast, when the experimentally challenged diabetic wounds delayed the expression of Krt16 to day 8, it failed to regulate the healing phases properly. This is because a lack of Krt16 leads to a failure to regulate the production of innate signals and an over-activation of the expression of cytokines and other regulators of skin barrier function [17]. Early activation of Krt16 expression after various types of injuries to the skin is therefore functionally relevant to the progression of cutaneous inflammation.

Krt16 was significantly inhibited in keratinocytes of diabetic wounds. In [27], the inhibition of Krt16 coincided with the induction of Smad2 in the corresponding epithelia. Thus, Krt16 was inhibited in keratinocytes in diabetic wounds due to the up-regulation of Smad2 expression, which is indeed increased in diabetes [28]. Furthermore, it was concluded that puerarin exerted its anti-diabetic effect on STZ-treated rats through the inhibition of the TGF-β1/Smad2 pathway [28]. It is likely that WP exerted its anti-diabetic effect on the STZ-treated rats in this study through the same pathway, and that this served to increase Krt16, which is important for keratinocyte migration and epidermal hyperplasia. This suggested mechanism could explain the normalizing effect of WP in the diabetic wounds mediated by Krt16. Thus, loss of Krt16 at the optimal time checkpoint in diabetic wounds may impair the inflammatory stage resulting in inappropriate immune responses [17].

## Conclusions

Our data confirms a role for Krt16 in the improvement of the inflammatory phase in the diabetic wounds, thereby leading to successful healing. In addition, WP was able to stimulate Hsp72 expression in early phases of wound healing in diabetic rats, resulting in a more normal healing process. From a therapeutic point of view, it is important to determine a protein by which Hsp72 and Krt16 can be induced. However, further studies are also warranted to investigate the role of WP as a potential future pharmacological approach to combat metabolic diseases.

## Abbreviations

CMC: carboxymethyl cellulose
Hsp72: heat shock protein-72
HE: hematoxylin and eosin
ROS: reactive oxygen species
Krt16: keratin16
STZ: streptozotocin
TNF-a: tumor necrosis factor-alpha
T1D: type 1 diabetes
T2D: type 2 diabetes
WP: whey protein
